# Dissecting tumor heterogeneity in colorectal cancer: uncovering the role of BCL2L1^+^ cells through single-cell analysis

**DOI:** 10.3389/fimmu.2026.1742767

**Published:** 2026-03-25

**Authors:** Guangsheng Zhu, Ya Liu, Yunxuan Shi, Nuan Qian, Chengcheng Song, Xu Liu, Zhikai Xiahou, Zhiguo Xiong, Junjie Hu

**Affiliations:** 1 Department of Gastrointestinal Surgery, Hubei Cancer Hospital, Tongji Medical College, Huazhong University of Science and Technology, Wuhan, China; 2 Hubei Provincial Clinical Research Center for Colorectal Cancer, Wuhan, China; 3 Department of Rheumatology and Immunology, Traditional Chinese and Western Medicine Hospital of Wuhan, Tongji Medical College, Huazhong University of Science and Technology, Wuhan, China; 4 First Clinical Medical College, Shandong University of Traditional Chinese Medicine, Jinan, China; 5 School of Life Sciences and Technology, Henan Medical University, Xinxiang, China; 6 Clinic Medicine, Henan Medical University, Xinxiang, China; 7 China Institute of Sport and Health Science, Beijing Sport University, Beijing, China

**Keywords:** BCL2L1+ subtype, CRC, immune evasion, liver metastasis, scRNA-seq

## Abstract

**Background:**

Colorectal cancer (CRC) ranks among the most prevalent gastrointestinal malignancies with liver metastasis being the primary cause of CRC-related death. Although surgical and chemotherapeutic interventions continue to improve, patients with hepatic metastases frequently experience recurrence and limited treatment benefits. Liver metastasis is driven by tumor heterogeneity and immune evasion. Therefore, defining the cellular composition of CRC liver metastases may help identify new therapeutic targets.

**Methods:**

Primary CRC and liver metastasis samples were analyzed by single-cell RNA sequencing (scRNA-seq). Seurat was used for quality control, dimensionality reduction, clustering, and cell annotation, and Harmony corrected batch effects. Differential expression with GO, KEGG, and GSEA was performed for enrichment. Copy number variation analysis using inferCNV (v1.1) distinguished malignant from non-malignant cells, with smooth muscle cells (SMCs) and epithelial cells (EPCs) as reference populations. CytoTRACE and Slingshot characterized tumor differentiation trajectories, while CellChat and pySCENIC constructed cell communication and transcriptional regulatory networks. The key factor *CEBPG* was validated by *in vitro* functional experiments. Statistical analyses were conducted in R and Python.

**Results:**

scRNA-seq identified five CRC tumor cell subtypes, among which the C4 *BCL2L1^+^* tumor cells (TCs)subtype was predominantly enriched in liver metastases and displayed enhanced proliferation, metabolic reprogramming, and anti-apoptotic activity. Furthermore, transcription factor analysis suggested that *CEBPG* might regulate *BCL2L1* expression to promote tumor survival and migration. Subsequent *CEBPG* silencing markedly suppressed CRC cell proliferation and invasion. In addition, a BTRS model derived from the C4 *BCL2L1^+^* TCs subtype effectively stratified patient prognosis, as the high-risk group exhibited elevated expression of immune escape–related genes and impaired immune function.

**Conclusion:**

This study revealed that the C4 *BCL2L1^+^* TCs subtype might drive CRC progression by promoting metabolic adaptation and immune evasion. In addition, *CEBPG* functioned as a key regulatory factor that increased tumor malignancy through *BCL2L1*-mediated survival pathways to some extent. The BTRS model reflected the molecular and immune heterogeneity of CRC and provided a framework for clinical risk stratification and personalized therapy. In summary, this work provides a comprehensive mechanistic framework linking metabolic adaptation, immune escape, and the progression and metastasis of CRC, and identifies potential therapeutic targets.

## Introduction

Colorectal cancer (CRC) accounts for approximately 10% of all cancer cases, ranking third in incidence worldwide. In Asia, the five-year prevalence of CRC is 55.60 per 100,000 people, and it is a leading cause of cancer-related deaths (1–4). Since about 90% of CRC fatalities are caused by tumor metastases, understanding metastatic mechanisms is critical (5). It has been reported that there are significant differences in the 5-year overall survival rate (OS) among different stages: approximately 90% for stage I, 70% for stage II, 58% for stage III, and less than 25% for stage IV (6). Notably, for patients with distant metastases, the 5-year OS further drops below 15% (7, 8). Although advances in surgery, chemotherapy, and early screening improve outcomes, CRC remains difficult to manage due to its strong metastatic nature and molecular diversity (9). Furthermore, while immunotherapy, particularly immune checkpoint inhibition, has achieved breakthroughs in several cancers, its effectiveness in CRC is limited to a subset of patients with POLE/POLD1 mutations or dMMR/MSI-H profiles (10, 11). Conversely, most microsatellite-stable (MSS) CRCs exhibit poor therapeutic responses (12). These challenges-heterogeneity, recurrence, and resistance-are increasingly recognized as being driven by the tumor microenvironment (TME) (13).

TME serves as a critical determinant of CRC initiation, metastasis, and therapeutic resistance (14). It is composed of cancer cells alongside multiple immune and stromal populations-such as tumor-associated macrophages, epithelial and endothelial cells, and natural killer (NK) cells-forming a dynamic molecular network (15). Within this complex milieu, cancer cells orchestrate communication by releasing chemokines and exosomes that regulate immune cell recruitment and by remodeling the extracellular matrix to facilitate tumor progression (16). Therefore, exploring the molecular mechanisms driving tumor-TME interactions may reveal valuable therapeutic targets for innovative CRC interventions (17).

Single-cell RNA sequencing (scRNA-seq) technology enables precise characterization of tumor heterogeneity and the diverse cellular components of the TME (18). Although genomic variations between primary CRC and its liver metastases are relatively minor, their cellular landscapes differ profoundly. Specifically, liver metastatic lesions display distinct T cells proportions compared with other sites, reflecting possible immune suppression or exclusion. This immune heterogeneity suggests impaired local immunity in metastatic tumors and may explain the limited response to immunotherapy (5, 19).

To dissect how immune and stromal cells cooperatively modulate the CRC microenvironment, we performed a systematic scRNA-seq analysis across primary and liver metastatic CRC samples. Specifically, we investigated how distinct tumor cell subtypes interact with immune and stromal components by integrating cell-type composition analysis, transcriptional profiling, and cell-cell communication inference. We identified the C4 *BCL2L1^+^* TCs subtype, present in both primary and liver metastatic CRC, as a critical component enriched in metastatic lesions. This subtype is characterized by high stemness and elevated oxidative phosphorylation (OXPHOS) activity, which may provide metabolic resilience and promote metastatic adaptation (20). Network analysis reveals that C4 *BCL2L1^+^* TCs serve as communication hubs within the tumor ecosystem. Functional validation via *CEBPG* knockdown confirms its regulatory role and potential as a therapeutic target. Additionally, we established a prognostic model based on cancer cell subtype characteristics and their immune interactions, offering mechanistic insight and predictive value for CRC management.

## Materials and methods

### Data acquisition for CRC

scRNA-seq data of CRC were collected from the Gene Expression Omnibus (GEO) database (https://www.ncbi.nlm.nih.gov/geo/) under accession number GSE231559. The dataset provided comprehensive gene expression profiles and clinical baseline information for CRC patients. According to tissue origin, samples were divided into primary and liver metastatic CRC to enable subsequent comparative analyses. As these data were derived from publicly available resources, ethical approval and informed consent are not required.

### Single-cell RNA sequencing data processing and analysis

For in-depth analysis, gene expression data were loaded into R and processed through the Seurat package. Rigorous quality controlled eliminates low-quality cells, retaining only those with nFeature values between 300 and 5,000 and nCount between 500 and 50,000. Additionally, mitochondrial gene expression was restricted to under 25% of the total reads, and red blood cell–related genes comprised less than 5% of the total. The dataset was then normalized using NormalizeData and refined with FindVariableFeatures to obtain the top 2,000 variable genes. ScaleData standardized the data, paving the way for principal component analysis. Harmony corrected batch effects across samples. Next, FindClusters performed cell clustering, and dimensionality reduction was achieved via UMAP using the top 30 principal components (21, 22).

### Cell type identification

Cell clusters were first identified using the FindClusters and FindNeighbors functions in Seurat. Furthermore, the FindAllMarkers function applied the Wilcoxon rank-sum test to identify differentially expressed genes (DEGs) among various clusters, thereby enabling analysis of CRC cellular heterogeneity (23, 24). The average expression levels of marker genes were then calculated to annotate distinct cell subtype (25). Based on information from the CellMarker database (http://xteam.xbio.top/CellMarker/) and previously published literature, we determined the corresponding marker genes. Manual curation also played an essential role in ensuring annotation accuracy. Therefore, the application of this integrative approach enhanced the reliability of our results, demonstrating its effectiveness in improving data quality (26).

### Enrichment analysis

Functional enrichment analysis of Kyoto Encyclopedia of Genes and Genomes (KEGG) pathways and Gene Ontology (GO) terms was performed using the clusterProfiler R package (27). To further explore pathway-level differences among cell types, Gene Set Enrichment Analysis (GSEA) was employed to evaluate the enrichment DEGs across multiple cellular subsets (28–30). Statistical significance for enrichment analyses was determined using a threshold of p < 0.05. The significance levels were defined as follows: ns, P > 0.05; P < 0.05; *P < 0.01; and **P < 0.001.

### InferCNV analysis

Copy number variation (CNV) analysis was carried out on scRNA-seq data using the inferCNV R package (https://github.com/broadinstitute/inferCNV) (31, 32). This analysis relied on relative gene expression levels and chromosomal location information to infer CNV states at the single-cell level, enabling discrimination between malignant tumor cells and normal counterparts. For this purpose, smooth muscle cells (SMCs) were selected as the reference population because of their genomic stability in CRC tissues. As terminally differentiated stromal cells, SMCs are generally non-malignant and exhibit minimal copy number alterations. Their distinct developmental origin and biological functions compared with epithelial tumor cells further reduce potential bias introduced by tumor-associated genomic aberrations. In addition, SMCs showed clear annotation and low transcriptional variability in our dataset, supporting their suitability as a reference baseline for accurate CNV inference in epithelial cells (EPCs).

### Trajectory analysis

We used CytoTRACE to rank malignant CRC cells in order to understand their developmental progression and differentiation (33), which estimates cellular differentiation states based on transcriptional complexity, with higher scores indicating a more primitive and less differentiated transcriptional profile. Then, we employed Slingshot to generate trajectory maps of tumor cell subtypes, while pseudotime analysis captures the temporal features of their differentiation (34). The getLineages and getCurves functions helped us infer the developmental paths of each subtype.

### Cell-cell communication

To examine cell-cell interactions, we utilized CellChat to forecast potential communication among various cell types (35). This tool provided quantitative insights and supports scRNA-seq data analysis (36, 37). We measured changes in interaction strength with netVisual_diffInteraction and applied the built-in pattern recognition to identify unique communication modes.

### pySCENIC analysis

For regulatory network analysis, we implemented pySCENIC to build gene regulatory networks and assess the activity of enriched transcription factors (36). This analysis allowed us to uncover transcription factors (TFs) that were active in specific cell states, investigate their regulatory mechanisms, and study the resulting gene expression changes, thereby clarifying the molecular basis of cell state transitions (38).

### Development and validation of prognostic prediction models

Relevant datasets were obtained from the TCGA database, and univariate Cox regression analysis was performed to identify 14 genes significantly associated with prognosis. To reduce potential gene multicollinearity, candidate genes were further screened using LASSO regression (39). Subsequently, multivariate Cox regression analysis identified 11 key prognostic genes for constructing a risk prediction model (40). The risk score for each sample was calculated using the following formula:


$$\text{Risk score=}\left({\text{Gene}}_{1}\;{\text{expression×Coefficient}}_{1}\right)+\left({\text{Gene}}_{2}\;{\text{expression×Coefficient}}_{2}\right)\text{ + … + }\left({\text{Gene}}_{\text{n}}{\text{ expression×Coefficient}}_{\text{n}}\right).$$

Patients were then stratified into low BTRS and high BTRS groups based on the optimal threshold determined by the improved surv_cutpoint function. Survival differences between subgroups were analyzed using the Survival package, and survival curves were plotted with ggsurvplot. Model accuracy was assessed through ROC curve analysis using the timeROC package. Furthermore, to determine whether the risk score served as an independent prognostic factor, multivariate Cox regression analysis was conducted.

### Analysis of the immune microenvironment

Immune-related scores of 22 immune cell subsets were calculated via the CIBERSORT R package. Moreover, three complementary algorithms-CIBERSORT, ESTIMATE, and Xcell-were applied to systematically evaluate the immune contexture of each patient. We analyzed both the differential infiltration levels of immune cells and the expression of immune checkpoint-associated genes, thereby capturing the dynamic immune features. Finally, comprehensive correlation analyses among immune parameters, model genes, and risk scores were performed to ensure the robustness of our findings (41).

### Drug sensitive analysis

We utilized the pRRophetic R package to estimate the half-maximal inhibitory concentration (IC50) values of CRC-associated therapeutic drugs across distinct risk groups. Moreover, drug sensitivity datasets were retrieved from the Genomics of Drug Sensitivity in Cancer database (https://www.cancerrxgene.org/) to serve as reference files. This integration allowed for a more accurate alignment between our analytical outcomes and clinical drug response patterns (42, 43).

### Cell culture

SW48 and SNU-81 cell lines were derived from colorectal adenocarcinoma. SW48 cells were maintained in DMEM supplemented with 10% fetal bovine serum (FBS) and 1% penicillin–streptomycin, while SNU-81 cells were cultured in RPMI-1640 medium under identical conditions. All cells were incubated at 37 °C in a humidified atmosphere containing 5% CO_2_ (44–46).

### siRNA transfection

Cells were seeded at a density of 2^5^ cells per well in six-well plates. After 24 hours, transfection was performed using Lipofectamine RNAiMAX with *CEBPG*-specific small interfering RNA (siRNA, GenePharma) at a final concentration of 20 μM. After another 24 hours, the cells were collected for subsequent analyses. The *CEBPG* siRNA sequences were as follows: siRNA1, GCGACAAUGCAGGACAGUA; siRNA2, CAACGCCGAGAGAGGAACA.

### Western blot

Cells were transfected at approximately 70% confluence and harvested 24 h after transfection. Cells were lysed in RIPA buffer, and the lysates were centrifuged at 12,000 rpm for 15 min at 4 °C to remove cellular debris. The lysates were centrifuged at 12,000 rpm for 15 minutes, and the supernatants were separated by SDS-PAGE. Proteins were then transferred to PVDF membranes and blocked with 5% bovine serum albumin (BSA) for 1.5 hours at room temperature to minimize non-specific binding. Subsequently, membranes were incubated overnight at 4 °C with anti-*CEBPG* antibody, followed by secondary antibody incubation for 1 hour at room temperature. Protein bands were visualized using enhanced chemiluminescence reagents (47).

### Quantitative real-time polymerase chain reaction

The TRIzol reagent (Thermo Fisher Scientific, Waltham, MA, USA) was used to extract total RNA in accordance with the manufacturer’s instructions. The PrimeScript™ RT kit (TaKaRa, Tokyo, Japan) was used to reverse-transcribe 500 ng of total RNA into cDNA. Next, using SYBR^®^ Premix Ex TaqTM (TaKaRa) on an ABI ViiA™ 7 real-time fluorescence detection system (Applied Biosystems, Indianapolis, IN, USA), quantitative real-time PCR (qRT-PCR) was carried out (23). The specific design of the *CEBPG* primers was as follows: Forward primer: 5′-CCCATGGATCGAAACAGTGAC-3′; Reverse primer: 5′-CTGCAGTGTGTCTTGTGCTT-3′ (48).

### Cell viability identification

Cells were seeded in 96-well plates at a density of 1×10³ cells per well and incubated for 8 hours (49). Each well then received 100 μL of detection reagent and was incubated for 1 hour. Absorbance was measured at 450 nm every day for four consecutive days, and OD450 values were used to generate cell growth curves.

### Transwell assay

Before the experiment, cells were serum-starved for 24 hours. A suspension of cells mixed with Matrigel (BD Biosciences, USA) was added to the upper chamber of a Transwell insert, while the lower chamber contained serum-rich medium to establish a chemotactic gradient. After incubation for 48 hours, cells were fixed with 4% paraformaldehyde and stained with crystal violet to assess their migratory and invasive capacities (50).

### Wound healing assay

Cells were seeded into six-well plates and grown to full confluence. A sterile 200 μL pipette tip was used to make a linear scratch across the monolayer. The cells were then washed with PBS to remove debris and cultured in serum-free medium. Images were captured at 0 and 48 hours to record wound closure, and the migration rate was quantified using ImageJ software.

### 5-ethynyl-2’-deoxyuridine proliferation assay

Transfected SW48 and SNU-81 cells were seeded into six-well plates at a density of 5×10³ cells per well and incubated for 24 hours. Cells were then incubated for 2 hours in culture medium containing the EdU working solution (51), fixed with 4% paraformaldehyde, and permeabilized with 2 mg/mL glycine and 0.5% Triton X-100. Subsequently, the cells were stained with 1× Apollo and Hoechst solutions for 30 minutes. Fluorescence microscopy was used to observe and quantify cell proliferation.

### Statistical analysis

All statistical analyses were conducted using R and Python software. P-values were calculated to determine statistical significance, which was denoted as P < 0.05, *P < 0.01, **P < 0.001, and ***P < 0.0001 (52); ns indicates no statistical difference. These statistical methods and significance thresholds ensured the robustness and reliability of the experimental findings (53, 54).

## Results

### scRNA-seq analyzes CRC cell heterogeneity

**Figure 1:**
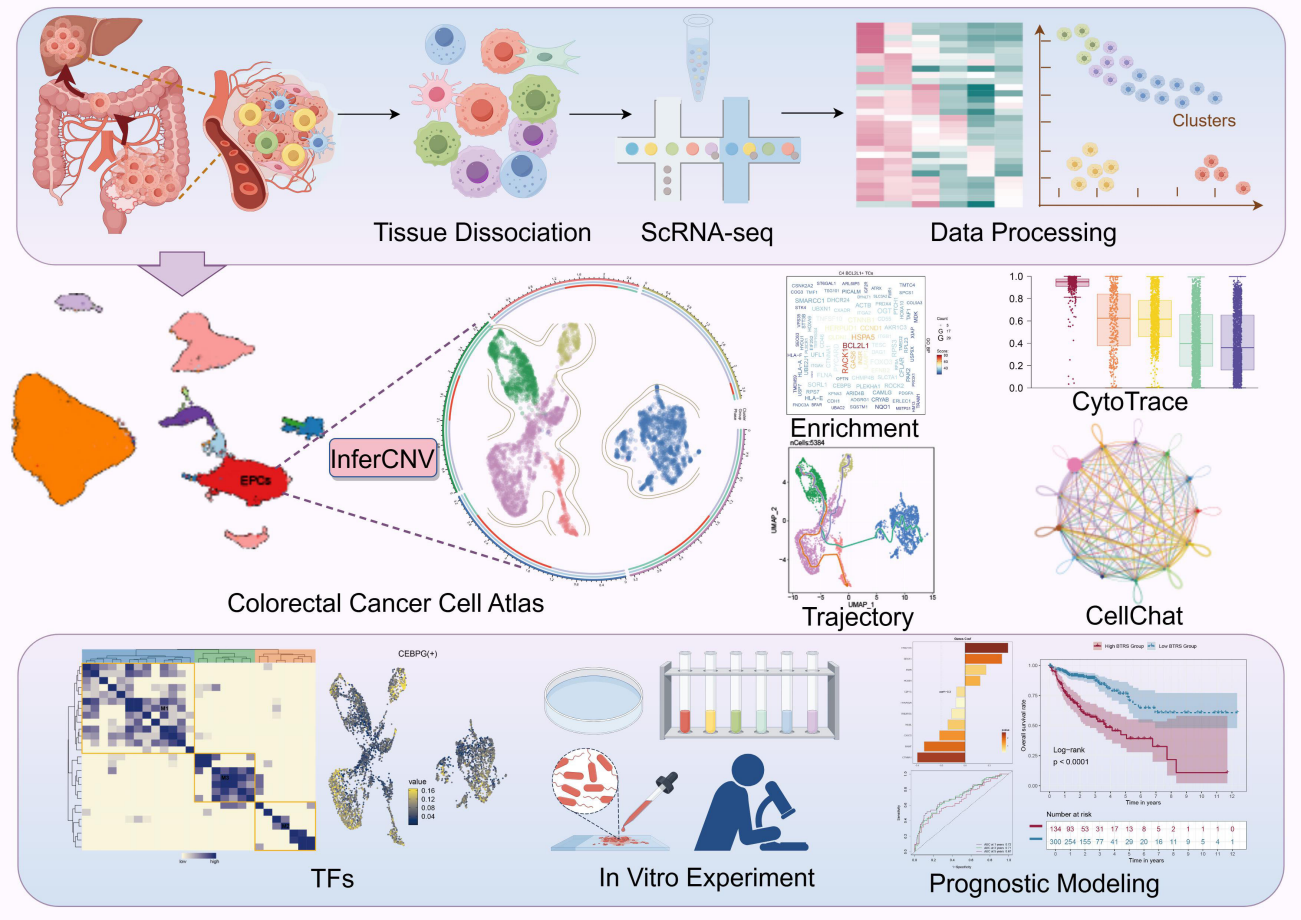
Workflow of single-cell sequencing analysis for the GSE231559 dataset. Single-cell analysis of CRC tissue heterogeneity.

**Figure 2:**
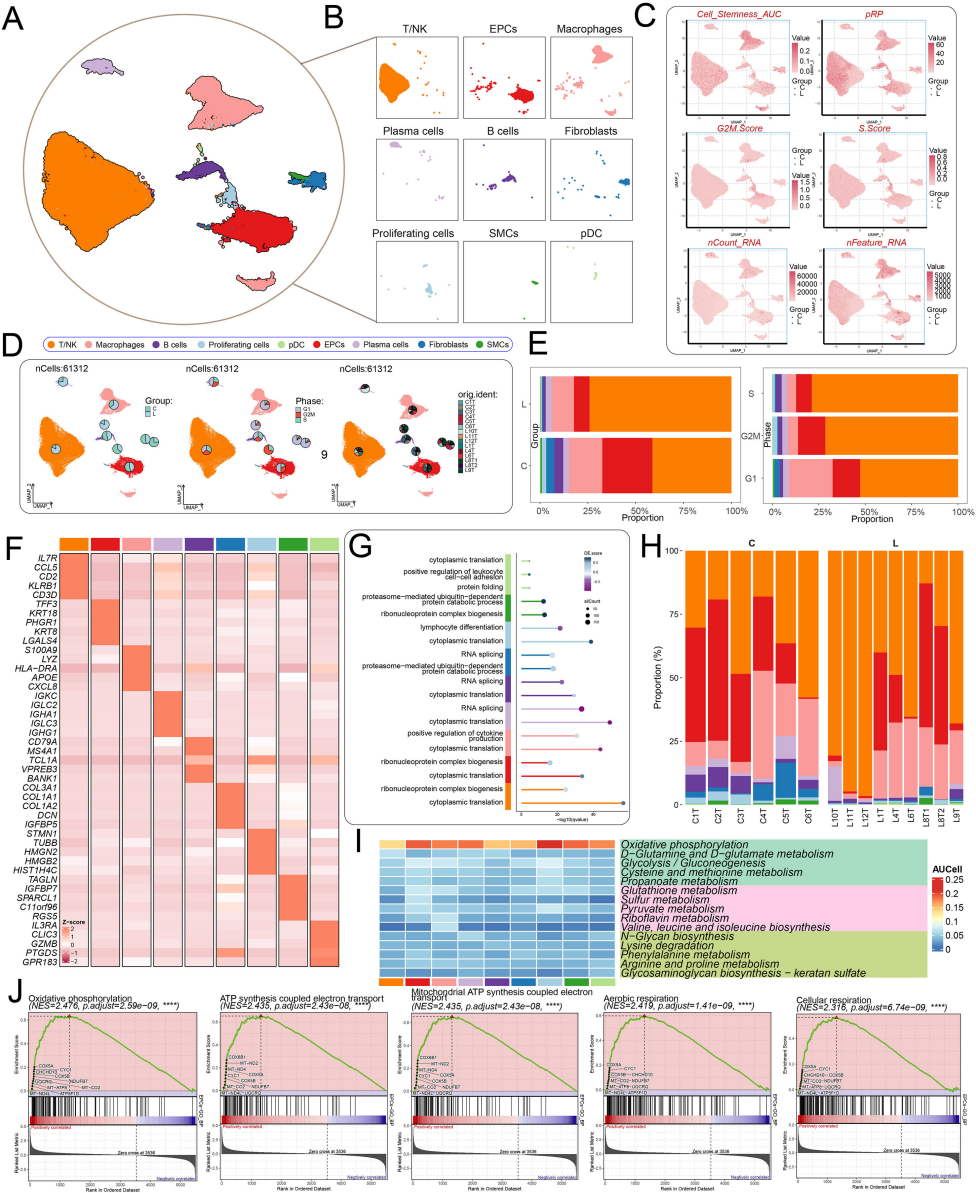
Single-cell analysis of CRC tissue heterogeneity. **(A)** Dimensionality reduction and clustering of cells from selected tissues, with annotated cell types. **(B)** Facet plots illustrating the distribution characteristics of each cell type. **(C)** UMAP plots showing the distribution of cell types based on Cell-Stemness-AUC, pRP, G2/M score, S score, nCount-RNA, and nFeature-RNA. **(D)** UMAP plots with overlaid pie charts illustrating the proportions of sample origin, cell cycle phase, and tissue classification across different cell types. **(E)** Stacked bar plots representing the relative proportions of each cell type within groups and cell cycles. **(F)** Heatmaps displaying the top five marker genes for each cell type. **(G)** Comparative analysis of biological function enrichment among cell types. **(H)** Stacked bar plots showing the proportions of each cell type across patients from different sample sources. **(I)** Heatmaps showing AUCell scores of the top five metabolic pathways in the identified cell types. **(J)** GSEA enrichment analysis highlighting pathways positively enriched in EPCs. ****P < 0.0001.

**Figure 1** illustrated the overall experimental workflow. Through unsupervised clustering and UMAP visualization of scRNA-seq data, the study revealed the cellular cluster architecture within tissue samples (**Figure 2A**). By integrating classical marker gene expression profiles, the clusters were annotated as T/NK cells, EPCs, macrophages, plasma cells, B cells, fibroblasts, proliferating cells, SMCs, and plasmacytoid dendritic cells (**Figure 2B**). In the diagram, group C represented primary tumor, while group L represented liver metastases. Further analyses suggested that these cell types exhibited distinct differences in stemness, proliferative capacity, and cell cycle distribution. In particular, proliferating cells displayed strong proliferative activity, whereas EPCs showed moderate potential (**Figure 2C**). Moreover, variations in the distribution of cells across the G1, S, and G2/M phases likely influenced tumor growth dynamics (**Figure 2D**). As shown in the stacked bar plot, T/NK cells, EPCs and Macrophages constituted the predominant population in liver metastases and across all cell cycle (**Figure 2E**). The accuracy of cell annotations was further validated by a heatmap of representative marker genes. EPCs highly expressed *TFF3*, *KRT18*, *PHGR1*, *KRT8*, and *LGALS4*; macrophages expressed *S100A9*, *LYZ*, *HLA-DRA*, *APOE*, and *CXCL8*; proliferating cells expressed *STMN1*, *TUBB*, *HMGN2*, *HMGB2*, and *HIST1H4C*; and fibroblasts expressed *COL3A1*, *COL1A1*, *COL1A2*, *IGFBP5*, and *DCN* (**Figure 2F**). The enrichment analysis revealed that different cell types exhibited distinct functional differentiation in aspects such as protein metabolism, RNA processing, and immune function regulation, reflecting the specific biological functions of various cell populations in the tissue microenvironment (**Figure 2G**). Following CRC metastasis to the liver, the proportions of major cell populations shifted, leading to distinct alterations in the TME between primary and metastatic lesions (**Figure 2H**). Furthermore, metabolic pathway analysis suggested that EPCs positively regulated OXPHOS, ATP synthesis–coupled electron transport, and related pathways (**Figures 2I, J**). Finally, inferCNV analysis using SMCs as a reference. SMCs showed largely uniform copy number signals across chromosomes, consistent with a relatively stable genomic profile. By comparison, EPCs displayed frequent copy number changes, with broad regions of chromosomal gains and losses observed across multiple chromosomes. These distinct CNV patterns clearly separated EPCs from the reference population and were indicative of malignant EPCs features in CRC samples (**Supplementary Figure 1**). Consequently, EPCs were selected for further investigation into the intrinsic mechanisms underlying CRC progression.

### Molecular characterization of CRC tumor cell subtypes and enrichment analysis of their biological functions

**Figure 3:**
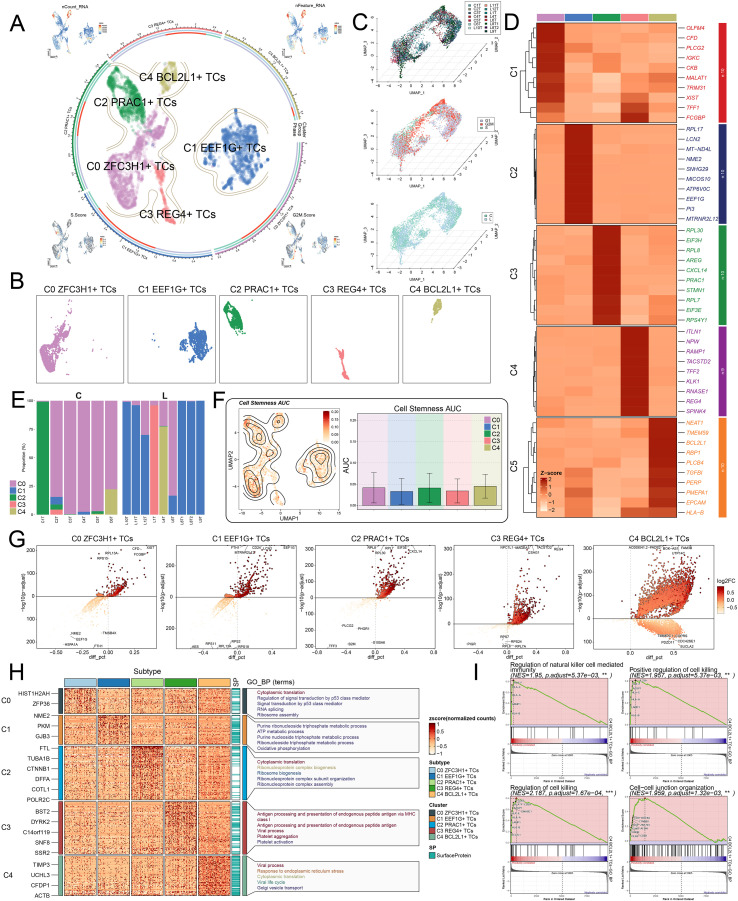
Subtype identification of CRC, differential gene analysis, and functional enrichment analysis. **(A)** Circular plot showing the clustering of five tumor cell subtypes identified in CRC. UMAP plots are arranged in four corners (clockwise from the upper left) showing the distribution of nCount-RNA, nFeature-RNA, G2/M score, and S score. **(B)** Facet plots illustrating the distribution of the five tumor cell subtypes. **(C)** UMAP plots depicting sample origin, cell cycle phase, and tissue classification of different cell subtypes in three dimensions. **(D)** Heatmap displaying the expression levels of the top 10 DEGs in the five tumor cell subtypes. **(E)** Stacked bar plot representing the proportion of each tumor cell subtype samples from different sample sources. **(F)** Contour plot showing stemness levels of the five tumor cell subtypes (left) and a bar plot comparing stemness differences among the subtypes (right). **(G)** Volcano plots illustrating significantly upregulated and downregulated genes in the five tumor cell subtypes. **(H)** Heatmap showing Gene Ontology (GO) biological process enrichment terms of DEGs in the five tumor cell subtypes. **(I)** GSEA enrichment analysis highlighting pathways positively enriched in the C4 *BCL2L1^+^* TCs subtype. **P < 0.01, ***P < 0.001.

To further elucidate the heterogeneity of CRC tumor cells, the study identified five distinct subtypes according to marker gene expression, namely C0 *ZFC3H1^+^* TCs, C1 *EEF1G^+^* TCs, C2 *PRAC1^+^* TCs, C3 *REG4^+^* TCs, and C4 *BCL2L1^+^* TCs (**Figures 3A, B**). Notably, among the identified tumor cell subtypes, the C4 *BCL2L1^+^* TCs subtype showed the most pronounced association with high-risk scores and adverse clinical features, particularly in liver metastasis samples. Subsequently, three-dimensional UMAP visualization was applied to depict their distribution and to compare subtype characteristics across different samples, patient groups, and cell cycle phases (**Figure 3C**). Furthermore, heat map analysis revealed unique gene expression profiles within each subtype. Specifically, the C0 *ZFC3H1^+^* TCs subtype highly expressed *OLFM4*, *CFD*, *PLCG2*, *IGKC*, and *CKB*; the C1 *EEF1G^+^* TCs subtype expressed *RPL17*, *LCN2*, *MT-ND4L*, *NME2*, and *SNHG29*; the C2 *PRAC1^+^* TCs subtype expressed *RPL30*, *EIF3H*, *RPL8*, *AREG*, and *CXCL14*; the C3 *REG4^+^* TCs subtype expressed *ITLN1*, *NPW*, *RAMP1*, *TACSTD2*, and *TFF2*; and the C4 *BCL2L1^+^* TCs subtype expressed *NEAT1*, *TMEM59*, *BCL2L1*, *RBP1*, and *PLCB4* (**Figure 3D**). The stacked bar plot further revealed differences in subtype composition between primary and metastatic CRC lesions (**Figure 3E**). Notably, the C4 *BCL2L1^+^* TCs subtype exhibited the highest stemness index, implying a potential association with increased tumor heterogeneity and a greater likelihood of recurrence (55) (**Figure 3F**). Volcano plots visualized upregulated and downregulated genes within each subtype (**Figure 3G**), while enrichment analysis suggested distinct biological functions. For instance, the C0 *ZFC3H1^+^* TCs subtype was linked to ribosome assembly and p53-mediated signaling, whereas the C1 *EEF1G^+^* TCs subtype was enriched in OXPHOS and ATP metabolism (56). The C2 *PRAC1^+^* TCs subtype was associated with ribonucleoprotein complex formation, the C3 *REG4^+^* TCs subtype with antigen presentation and platelet activation, and the C4 *BCL2L1^+^* TCs subtype with viral responses and ER stress (**Figure 3H**). GSEA further demonstrated that immune regulatory and cytotoxic gene sets were enriched in the C4 *BCL2L1^+^* TCs subtype (**Figure 3I**). Together, these findings suggested pronounced molecular and functional diversity among CRC tumor cell subtypes.

### Metabolic profiling and cell stemness reveal functional heterogeneity in CRC tumor cell subtypes

**Figure 4:**
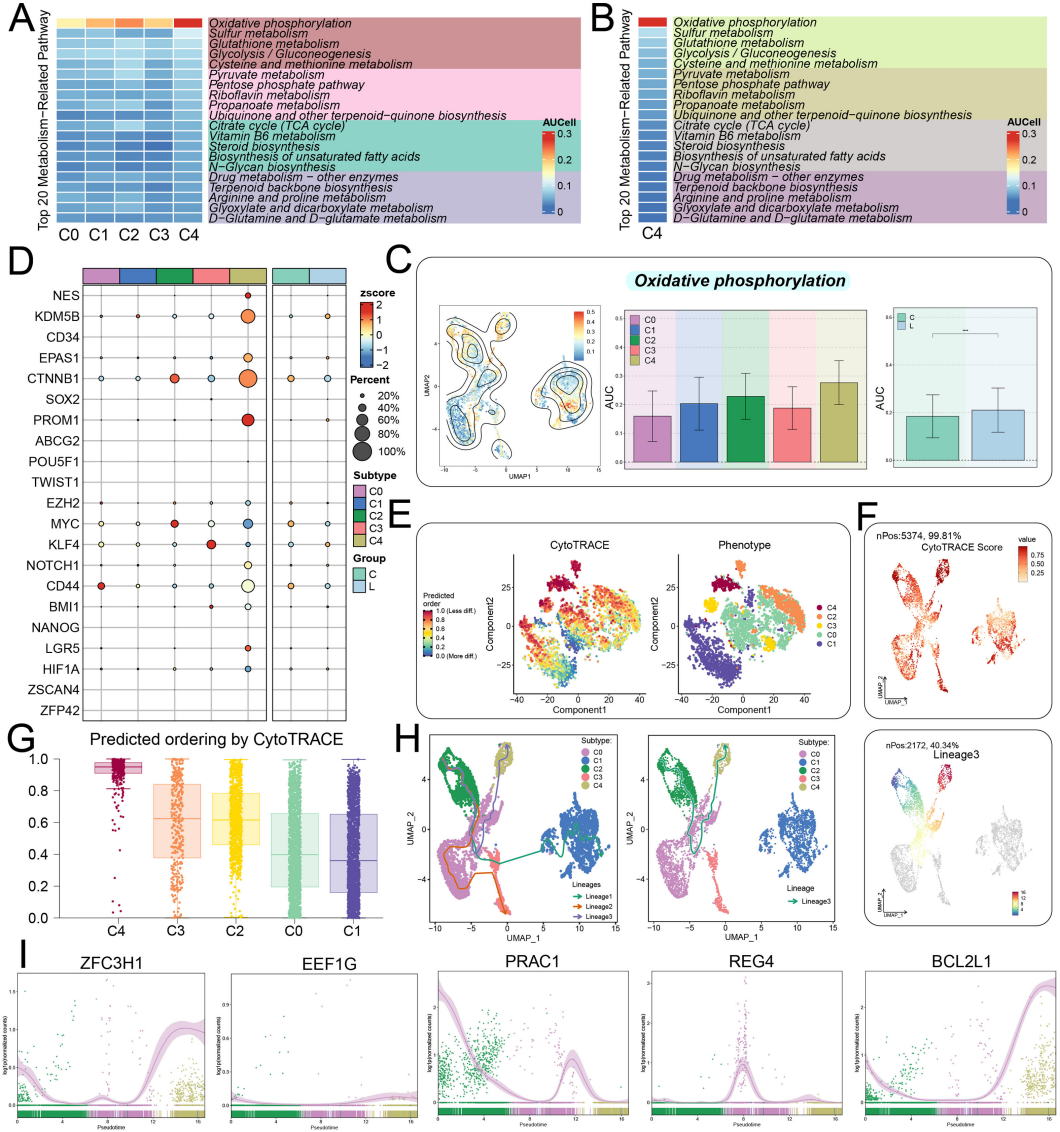
Metabolic, stemness, and pseudo-time analysis of tumor cell subtypes. **(A)** Heatmap showing AUCell scores of 20 metabolic pathways across five tumor cell subtypes. **(B)** Heatmap displaying AUCell scores of 20 metabolic pathways specifically in the C4 *BCL2L1^+^* TCs subtype. **(D)** Bubble plot illustrating differential expression of stemness-associated genes across tumor cell subtypes and tissue types. **(C)** Metabolic activity of oxidative phosphorylation in different cell subtypes and groups calculated using AUCell algorithms. **(E)** CytoTRACE plot predicting the ordered distribution of tumor cells, with color indicating stemness level (left), and a phenotype plot showing the distribution of tumor cell subtypes, colored by subtype (right). **(F)** UMAP plot showing CytoTRACE scores of the five tumor cell subtypes. **(G)** Ranking of tumor cell subtype stemness using CytoTRACE analysis. **(H)** UMAP plots depicting differentiation trajectories of Lineage1, Lineage2, and Lineage3 (left), with a focused view of Lineage3 (right). **(I)** Dynamic trend plots illustrating expression patterns of five named genes.

Metabolic pathway analysis compared the enrichment levels of multiple metabolic processes among the five CRC cell subtypes (**Figure 4A**). Interestingly, the most prominent expression in the C4 *BCL2L1^+^* TCs subtype is OXPHOS, which likely reflected its elevated energy demand and metabolic activity (57, 58) (**Figure 4B**). The distribution of OXPHOS across various cell populations was further illustrated through bar plots and UMAP visualization, both showing higher levels in the C4 *BCL2L1^+^* TCs subtype and liver metastasis samples (**Figure 4C**). In addition, bubble plots revealed significant gene expression differences among CRC subtypes and between primary and metastatic lesions (**Figure 4D**). Using CytoTRACE analysis, we observed that the C4 *BCL2L1^+^* TCs subtype exhibited the strongest stemness potential, ranking highest among all five subtypes (**Figure 4E**). According to the CytoTRACE-predicted differentiation hierarchy, the C4 *BCL2L1^+^* TCs subtype displayed a low degree of differentiation, whereas the C1 *EEF1G^+^* TCs subtype exhibited a high degree (**Figure 4F**). These findings were further validated by box plots, which showed that C4 *BCL2L1^+^* TCs scores were mainly concentrated above 0.8, while those of other subtypes were more dispersed (**Figure 4G**). Furthermore, differentiation trajectories reconstructed using Slingshot across three lineages illustrated CRC cell differentiation dynamics (**Figure 4H**). Finally, the dynamic trend plots indicated that the expression of *ZFC3H1*, *EEF1G*, *PRAC1*, *REG4*, and *BCL2L1*genes was unevenly distributed along the trajectories (**Figure 4I**).

### CellChat analysis reveals complex cellular communication networks and key signaling pathways in the CRC microenvironment

**Figure 5:**
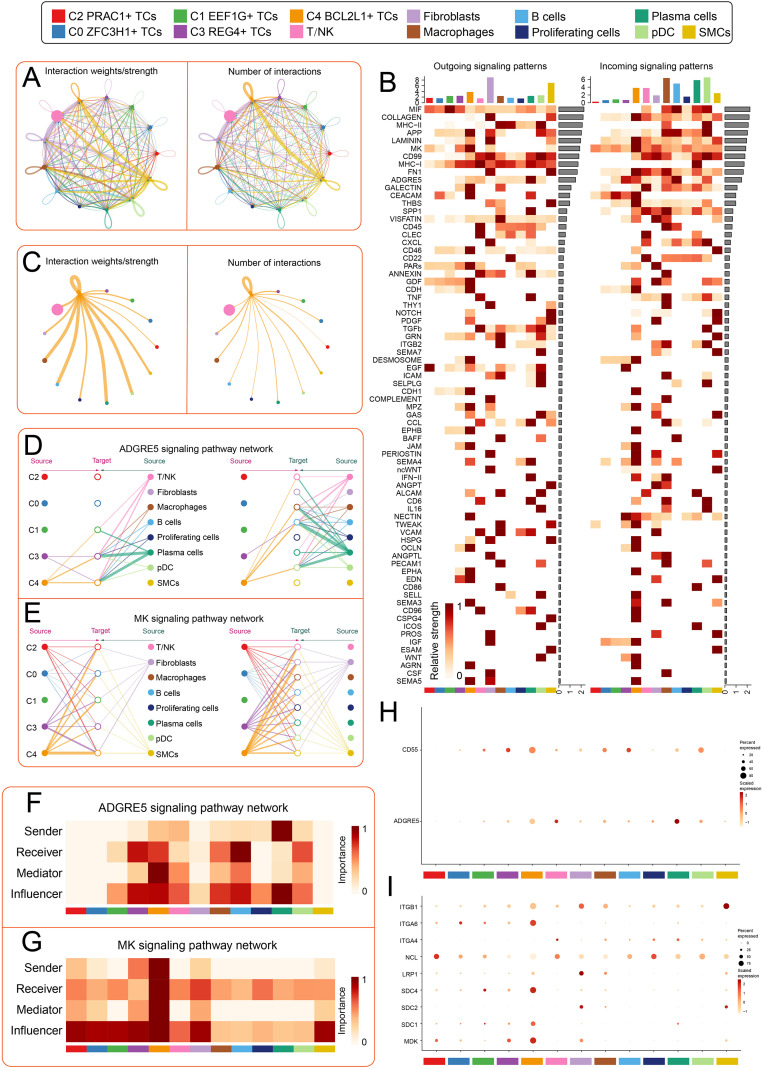
Analysis of tumor cell communication networks. **(A)** Circular plot showing interaction intensity (left) and number (right) among all cells in CRC. Circle size is proportional to the number of cells in each group, and edge width represents communication probability. **(B)** Bar plot comparing relative signal strength of tumor cell subtypes and other cell types in incoming and outgoing patterns. Heatmap visualizes the receiving intensity of various proteins within these communication patterns across the same cell subtypes. **(C)** Circular plot depicting interaction intensity (left) and number (right) between C4 *BCL2L1^+^* TCs malignant tumor cells (source) and other cell types. **(D, E)** Laminar plots showing interactions in the *ADGRE5* signaling pathway **(D)** and the *MK* signaling pathway **(E)**. **(F-G)** Heatmaps showing centrality scores of ligands and receptors in the *ADGRE5***(F)** and *MK***(G)** signaling pathways. **(H)** Bubble plot indicating potential interactions between C4 *BCL2L1^+^* TCs via *CD55-ADGRE5*. **(I)** Bubble plot comparing expression of key ligands and receptors in the *MK* signaling pathway across the five tumor cell subtypes and other cell types.

To further elucidate the crucial role of the C4 *BCL2L1^+^* TCs subtype in CRC, we employed CellChat analysis to explore its intrinsic intercellular communication mechanisms. As expected, T/NK cells represented the most abundant population, while the C4 *BCL2L1^+^* TCs subtype exhibited the greatest number and strength of interactions (**Figure 5A**). Subsequently, to obtain a more comprehensive view of the communication landscape, we analyzed both incoming and outgoing signaling patterns among the five CRC subtypes and other cell subtypes. Our results showed that *MIF*, *CD99*, and *MHC-I* pathways were markedly activated. Moreover, fibroblasts and SMCs mainly contributed to outgoing signals, whereas macrophages, pDCs, B cells, and plasma cells dominated the incoming communication (**Figure 5B**). When the C4 *BCL2L1^+^* TCs subtype was set as the central node, it displayed broad and strong connections with multiple cell subtypes (**Figure 5C**). In the hierarchical network, it was found to be closely associated with plasma cells through the *ADGRE5* pathway (**Figure 5D**). Moreover, within the *MK* signaling pathway, the C4 *BCL2L1^+^* TCs subtype exhibited strong interactions with SMCs and fibroblasts. (**Figure 5E**). Centrality analysis further revealed its dual role as an initiator and mediator in both the *ADGRE5* and *MK* pathways (**Figures 5F, G**). In addition, bubble plots validated the elevated expression of *ADGRE5*, *CD55*, and related proteins (**Figures 5H, I**). Collectively, these findings suggested that *ADGRE5* and *MK* signaling served as critical communication axes in CRC, by regulating the interaction among tumor cells, immune cells and stromal cells, it participates in the regulation of the immune microenvironment and promotes the malignant progression of tumors.

### Systematic analysis of TF regulation patterns in CRC cells

**Figure 6:**
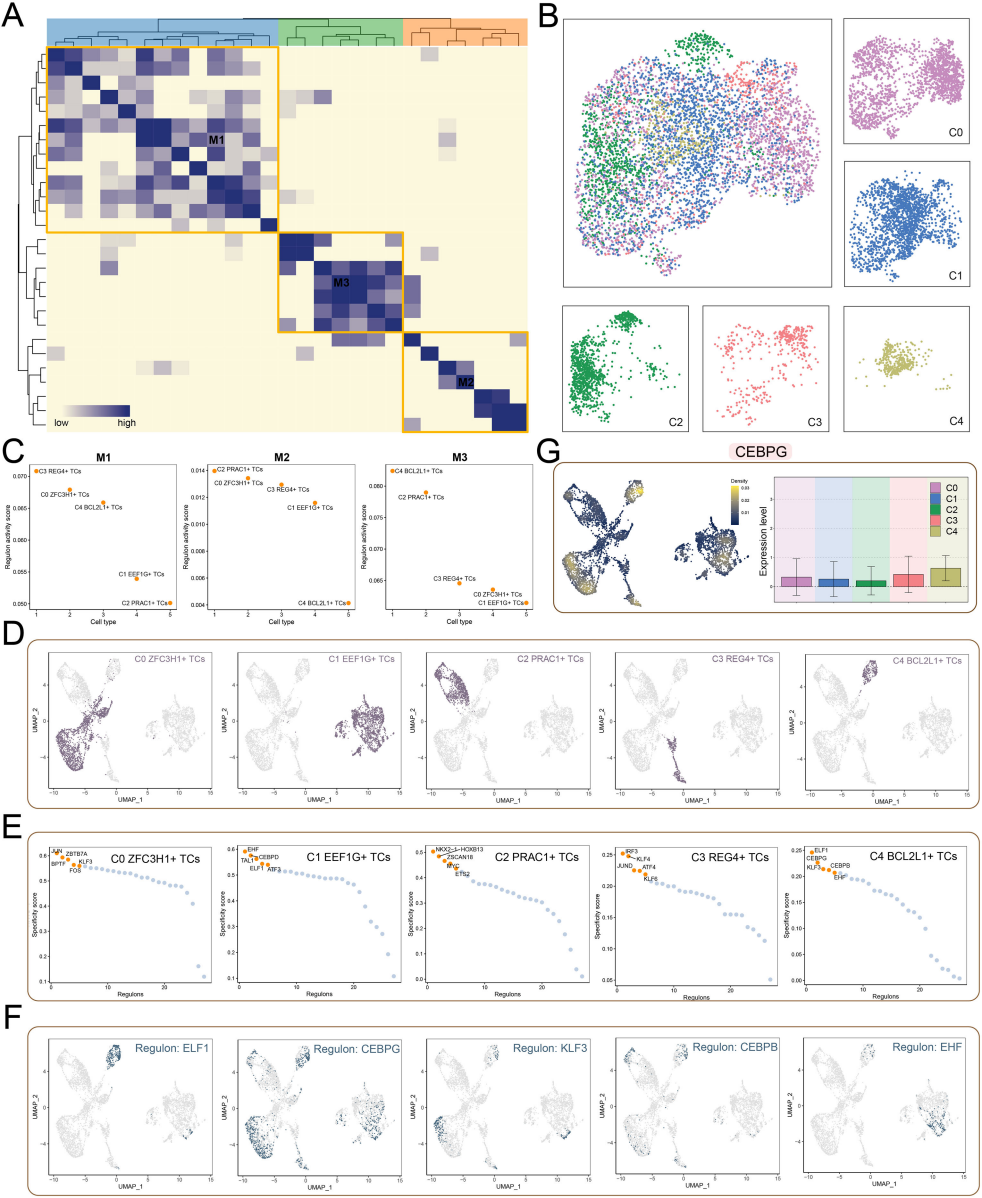
The TF regulatory mechanisms in the C4 *BCL2L1^+^* TCs subtype. **(A)** Based on the CSI matrix, TFs in tumor cell subtypes were grouped into three regulatory modules (M1, M2, M3). **(B)** UMAP plot showing the distribution of TFs across tumor cell subtypes, with surrounding facet plots illustrating the specific distribution within each subtype. **(C)** Regulatory effect scores of different tumor cell subtypes within the M1, M2, and M3 modules. **(D, E)** UMAP plots **(D)** and scatter plots **(E)** showing the distribution of TFs and activity score rankings of each subgroup, respectively. **(F)** UMAP plots displaying the top five TFs in C4 *BCL2L1^+^* TCs. **(G)** Density and expression of *CEBPG* in C4 *BCL2L1^+^* TCs visualized using UMAP (left) and bar plot (right), highlighting expression across tumor cell subtypes.

Based on the AUCell similarity scores, we classified transcription factors (TFs) with comparable expression and functions into three regulatory modules M1, M2, and M3, to further explore the TF network regulating the C4 *BCL2L1^+^* TCs subtype (**Figure 6A**). Moreover, UMAP visualization displayed the distribution of cell subtypes derived from scRNA-seq data, with each cell color-coded by its corresponding TF or cell type (**Figure 6B**). Subsequent analyses demonstrated that the C4 *BCL2L1^+^* TCs subtype exhibited the highest regulatory activity scores in module M3 (**Figure 6C**), which further confirmed its close relationship with proliferative activity. In addition, UMAP plots of each subtype highlighted distinct distributions of cell subtypes (**Figure 6D**). To elucidate the transcriptional regulatory basis of TCs we identified the top five TFs with the highest specificity scores across subtypes (**Figure 6E**). Accordingly, we compared the expression patterns of *ELF1*, *CEBPG*, *KLF3*, *CEBPB*, and *EHF* within the C4 *BCL2L1^+^* TCs subtype, and found that their expression profiles might play crucial roles in CRC initiation and progression (**Figure 6F**). Furthermore, *CEBPG*, a ferroptosis-associated TF, was significantly upregulated in CRC tissues, and its abnormal expression might have been linked to enhancer activation (59). Finally, density analysis revealed that *CEBPG* expression was particularly prominent in the C4 *BCL2L1^+^* TCs subtype (**Figure 6G**).

### Knockdown of CEBPG inhibits the proliferation, migration and invasion of CRC cells

**Figure 7:**
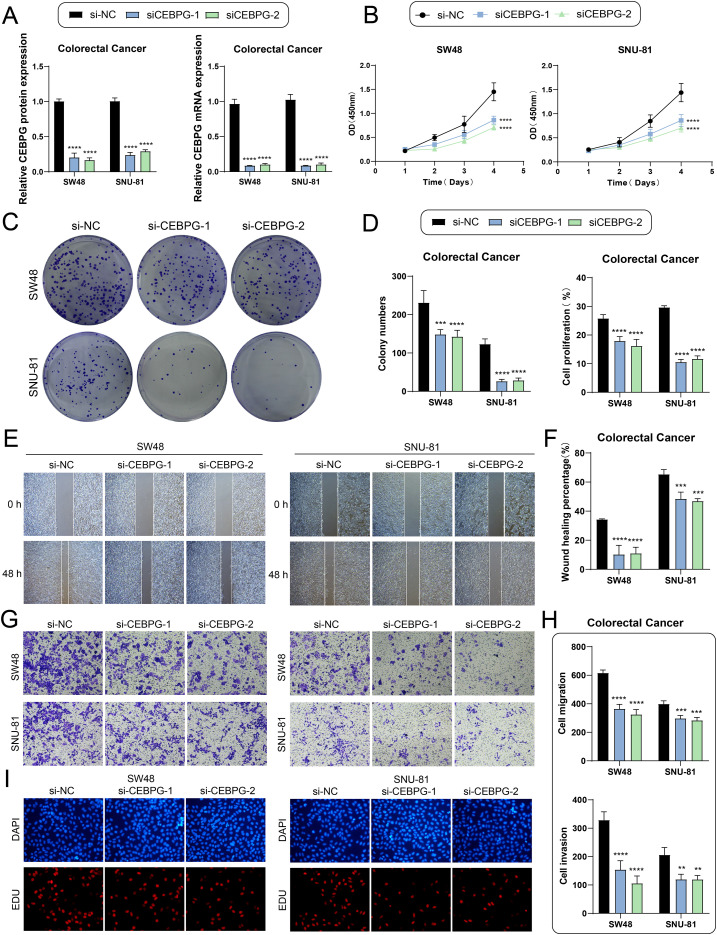
*In vitro* validation of *CEBPG* knockdown effects. **(A)** Bar plots showing protein (left) and mRNA (right) expression levels in SW48 and SNU-81 cell lines across si-NC, siCEBPG-1, and siCEBPG-2 groups. *CEBPG* knockdown significantly reduced both protein and mRNA expression. **(B)** CCK-8 assay results indicating a significant decrease in cell viability of SW48 and SNU-81 cells following *CEBPG* knockdown. **(C)** Colony formation assay demonstrating a marked reduction in colony numbers after *CEBPG* knockdown. **(D)** Bar plots quantifying the decrease in colony number (left) and proliferation ability (right) upon *CEBPG* knockdown. **(E)** Wound healing assay showing reduced migration ability of SW48 and SNU-81 cells after *CEBPG* knockdown. **(F)** Bar plot quantifying the decrease in cell migration following *CEBPG* knockdown. **(G)** Transwell assay demonstrating decreased migration and invasion of SW48 and SNU-81 cells after *CEBPG* knockdown. **(H)** Bar plots showing reduced cell migration (top) and invasion (bottom) after *CEBPG* knockdown. **(I)** EDU staining assay confirming the inhibitory effect of *CEBPG* knockdown on cell proliferation. **P < 0.01, ***P < 0.001, ****P < 0.0001.

Semi-quantitative analyses showed that both *CEBPG* protein and mRNA were expressed in SW48 and SNU-81 cell lines following si-NC, siCEBPG-1, and siCEBPG-2 treatments. As expected, *CEBPG* knockdown significantly reduced its expression at both protein and mRNA levels (**Figure 7A**). Furthermore, CCK-8 assays suggested that silencing *CEBPG* markedly and time-dependently decreased the viability of SW48 and SNU-81 cells (**Figure 7B**). Consistently, plate colony formation assays revealed that depletion of *CEBPG* significantly lowered colony numbers and suppressed proliferative capacity (**Figures 7C, D**). In addition, wound healing assays confirmed that *CEBPG* silencing markedly inhibited cell migration (**Figures 7E, F**), while Transwell assays further indicated that knockdown of *CEBPG* reduced both migratory and invasive abilities in SW48 and SNU-81 cells (**Figures 7G, H**). Moreover, EdU labeling assays showed that loss of *CEBPG* decreased the proportion of red-labeled proliferating cells, thereby restraining cell growth (**Figure 7I**). Taken together, these findings suggested that *CEBPG* played a crucial role in regulating CRC cell proliferation, migration, and invasion, highlighting its potential as a therapeutic target.

### Constructed the BTRS risk model for CRC

**Figure 8:**
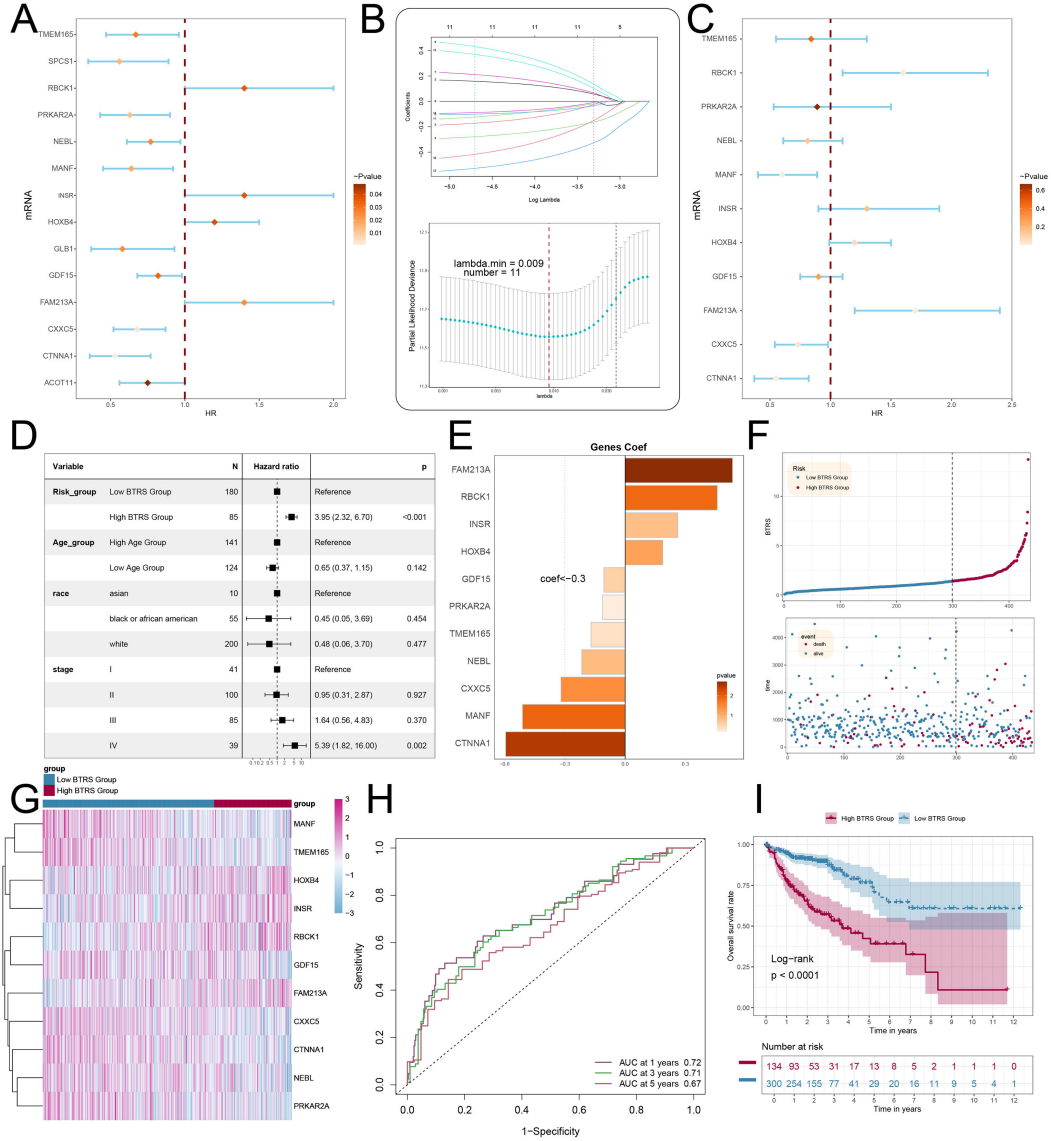
Construction and validation of the C4 *BCL2L1^+^* TCs risk score (BTRS) model. **(A)** Forest plot showing the top 14 genes identified by univariate Cox analysis associated with prognosis (HR < 1: protective factor; HR > 1: risk factor). **(B)** LASSO regression results: each line represents the coefficient of a gene with significant prognostic potential. Optimal parameters were determined via cross-validation (top), and the LASSO coefficient curve was generated using the optimal lambda (bottom). **(C)** Forest plot showing 11 prognostic genes identified by multivariate Cox analysis. **(D)** Multivariate Cox regression integrating the risk score with clinical factors (age, race, and tumor clinical stage). **(E)** Bar plot depicting the coefficients of the 11 prognostic genes. **(F)** Risk score distribution curves for the high and low BTRS groups (top), and scatter plots showing survival/death events over time (bottom). **(G)** Heatmap displaying differential expression of the 11 risk genes between the high and low BTRS groups. **(H)** ROC curves for survival prediction at 1 year (AUC = 0.72), 3 years (AUC = 0.71), and 5 years (AUC = 0.67). **(I)** Kaplan-Meier survival curve comparing survival between high and low BTRS expression groups.

Reliable molecular biomarkers are urgently needed for CRC prognosis. To tackle this challenge, we employed statistical approaches, including Cox regression and Lasso Cox regression, to construct a risk score model based on the C4 *BCL2L1^+^* TCs subtype. Initially, fourteen candidate prognostic genes were identified using univariate Cox regression (**Figure 8A**). Subsequently, Lasso Cox regression was applied to further refine the gene set and enhance model robustness (**Figure 8B**). Subsequently, multivariate Cox regression analysis confirmed 11 genes with independent prognostic significance. Among them, *RBCK1* and *FAM213A* may serve as risk factors, but without statistical significance (**Figure 8C**). Moreover, patients classified into the high BTRS group had a markedly higher risk of mortality compared with those in the low BTRS group (HR = 3.95, p < 0.001), reinforcing the prognostic power of the BTRS classification (**Figure 8D**). In contrast, most clinical parameters, including age and race, showed no significant associations with prognosis, whereas advanced disease stage (Stage IV) was significantly associated with poorer prognosis. Coefficient analysis further revealed that *FAM213A*, *RBCK1*, *INSR*, and *HOXB4* were positively correlated with poor outcomes (**Figure 8E**). Using the *BCL2L1^+^* subtype risk score cutoff value, patients were stratified to validate the clinical significance of the risk score (**Figure 8F**). Notably, higher expression levels of *RBCK1*, *INSR*, *HOXB4*, and *FAM213A* were observed in the high-risk group compared with controls (**Figure 8G**). Finally, ROC analysis yielded AUC values of 0.72, 0.71, and 0.67 for 1-, 3-, and 5-year survival, respectively, demonstrating satisfactory predictive performance (**Figure 8H**). Kaplan-Meier curves further confirmed significantly poorer survival in the high BTRS group (**Figure 8I**).

### Transcriptome analysis reveals prognostic-related molecules and immune heterogeneity in different BTRS subgroups in CRC

**Figure 9:**
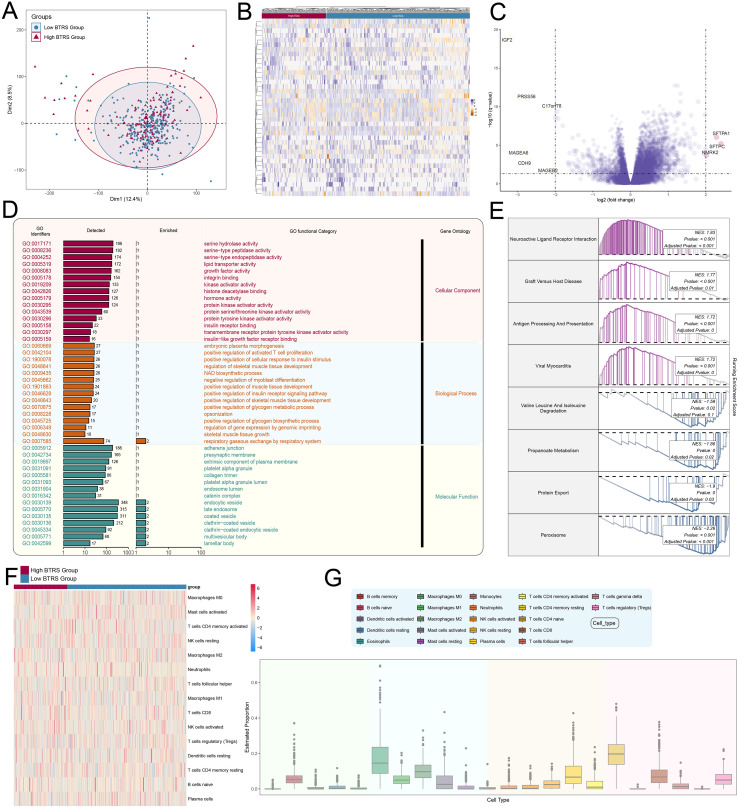
Functional enrichment and immune-related analysis of DEGs between high- and low-risk groups. **(A)** Scatter plot showing the distribution of genes along Dim1 and Dim2 in the high and low BTRS groups. **(B)** Heatmap displaying differential gene expression profiles between the high and low BTRS groups. **(C)** Volcano plot illustrating upregulated and downregulated genes between the high and low BTRS groups. **(D)** Bar plot presenting Gene Ontology (GO) enrichment analysis of DEGs between the high and low BTRS groups. **(E)** Comparison of GSEA enrichment results between the high and low BTRS groups. **(F)** Heatmap showing distinct immune patterns in the high and low BTRS groups. **(G)** Box plot depicting estimated proportions of 22 immune cell types across the high and low BTRS groups.

To assess overall differences in prognosis-related gene expression among CRC samples, we analyzed their distribution in principal component space derived from transcriptomic data. The PCA plot showed separation between high- and low-BTRS groups, indicating distinct transcriptional patterns between the two groups (**Figure 9A**). Consistently, the heatmap suggested distinct expression profiles across patient groups, where several genes were markedly upregulated or downregulated, suggesting a close relationship between these genes and CRC prognosis (**Figure 9B**). Furthermore, the volcano plot revealed significant upregulation of *SFTPA1*, *SFTPC*, and *NMRK2*, along with downregulation of *IGF2*, *PRSS56*, *MAGEA6*, *CDH9*, *MAGEB2*, and *C17orf78*, among which *IGF2* showed the greatest decrease (**Figure 9C**). To clarify the biological implications of these findings, we conducted GO enrichment analysis focusing on molecular function, biological process, and cellular component, which underscored the involvement of DEGs in key regulatory pathways (**Figure 9D**). Moreover, GSEA revealed that these genes participated in eight principal biological processes, including neuroactive ligand–receptor interaction, peroxisome, and protein export (**Figure 9E**). We subsequently examined immune cell infiltration differences between high and low BTRS groups and found that the high BTRS group displayed elevated immune activity and features suggestive of enhanced immunological engagement (**Figure 9F**). Although the high risk group exhibited higher immune-related scores, these metrics primarily reflect overall immune gene expression levels rather than the abundance of specific immune cell subsets. Additionally, we detected significant heterogeneity in immune infiltration, characterized by increased proportions of activated CD4 memory T cells and M0 macrophage subtypes (**Figure 9G**). Collectively, these findings provided compelling evidence for the involvement of these genes in CRC progression and immune modulation, highlighting their value as potential prognostic biomarkers.

### Thorough evaluation of drug sensitivity and immune infiltration using a risk score

**Figure 10:**
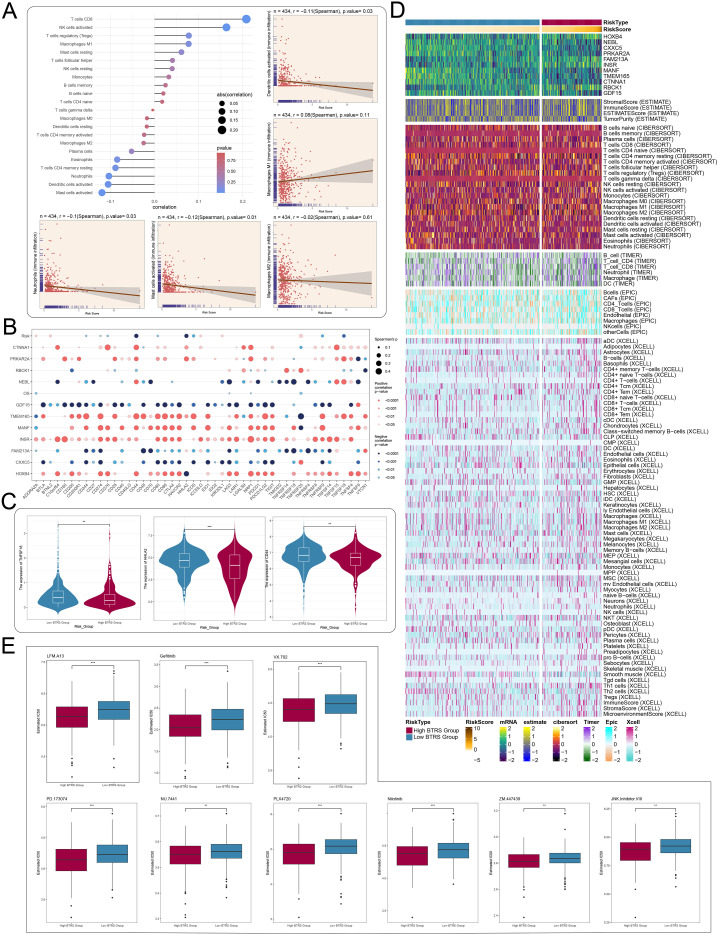
Immune infiltration characteristics and drug sensitivity analysis in different risk groups. **(A)** Lollipop plot showing relationships between immune cells and genes. Surrounding scatter plots display risk scores of five distinct cell types. **(B)** Bubble plot illustrating correlations among model genes, risk scores, overall survival (OS), and immune checkpoint-related genes. **(C)** Violin plots showing expression differences of *TNFSF18*, *HHLA2*, and *CD44* between high and low BTRS groups. **(D)** Heatmap highlighting differences in model gene expression, stromal score, immune score, ESTIMATE score, tumor purity, and immune cell infiltration levels (calculated using CIBERSORT and xCell) between high and low BTRS groups. **(E)** Box plots showing differences in IC50 values of various targeted drugs between high and low BTRS groups. **P < 0.01, ***P < 0.001.

We investigated the association between immune cell infiltration and the calculated risk score. The scatter plot showed that distinct immune cell types exhibited different correlations with the risk score. Specifically, activated NK cells and CD8^+^ T cells displayed strong positive associations, whereas activated mast cells and dendritic cells showed an inverse trend (**Figure 10A**). Bubble plot analysis further revealed that genes such as *GDF15*, *TMEM165*, *INSR*, and *FAM213A* showed correlations with multiple immune checkpoints (**Figure 10B**). To assess the potential for immunotherapy response, we compared *TNFSF18*, *HHLA2*, *CD44*, and signature scores between high and low BTRS groups, finding that the high-risk group had significantly higher levels of these indicators (**Figure 10C**). Heatmap analysis indicated that the high-risk group exhibited increased infiltration of CD4^+^ T cells and B cells, along with decreased NK cell and dendritic cell presence, while the low-risk group maintained a more uniform immune distribution, particularly in NK cells and macrophages. These findings suggested that immune infiltration patterns might impact CRC prognosis and reflect the tumor immune microenvironment (**Figure 10D**). Furthermore, drug sensitivity analysis showed that the high BTRS group exhibited lower predicted IC50 values for several compounds, including LFM.A13, gefitinib, and VX.702, based on in silico drug response modeling. Collectively, these results implied that immune microenvironment features and tumor characteristics were closely associated with therapeutic responses (**Figure 10E**).

## Discussion

Liver metastases were the primary cause the primary cause of morbidity and mortality in CRC (60). Therefore, there is an urgent clinical need for therapeutic strategies that could effectively inhibit liver metastasis. In this study, we systematically analyzed the cellular composition and functional characteristics of CRC primary tumors and liver metastases using scRNA-seq. ScRNA-seq analysis revealed that CRC tissues consisted of multiple distinct cell types. Furthermore, the clear clustering of these cells reflected the complex network of interactions within the TME, suggesting that each cell type played a specific role in metastasis, immune evasion, and tumor progression. We also observed considerable differences in proliferative activity and cell cycle distribution among the various cell subtypes. Additionally, metabolic profiling showed significant upregulation of OXPHOS and glycolysis pathways in epithelial and proliferating cells, indicating an increased energy demand (53).

EPCs play important roles in other cancers (61). For example, in glioblastoma, EPCs are mobilized to the tumor microenvironment by chemokines such as VEGF and SDF-1 secreted by the tumor, participating in tumor angiogenesis and promoting tumor growth. Therefore, to explore the specific mechanisms of action of EPCs in colorectal cancer, this study focuses on EPCs (62). Therefore, to explore the specific mechanisms of action of EPCs in colorectal cancer, this study focuses on EPCs. Studies showed that EPCs highly expressed the stemness gene *LGR5*. Furthermore, previous research indicated that *LGR5* acted as a key receptor in the Wnt signaling pathway and was mainly expressed in stem cells of organs such as the hair follicles and intestine. Consequently, it was widely regarded as a canonical marker of adult stem cells (63–65). *LGR5*-positive cell subtypes often exhibited features of cancer stem cells and were strongly linked to tumor progression, including metastasis, recurrence, and treatment resistance (66, 67). In addition, recent studies suggested that metastatic lesions displayed considerable plasticity, with many being initiated by *LGR5*-plastic cells that later reacquired stemness, highlighting the complex role of *LGR5 (*68–70).

Using unsupervised clustering combined with UMAP visualization, we identified five distinct cell subtypes. Among them, the C0 *ZFC3H1^+^* TCs subtype highly expressed *OLFM4*, which had previously been regarded as a stemness-related marker in the intestinal epithelium (71) and was often linked to differentiation markers in CRC; however, recent studies produced conflicting results regarding its direct role in stemness or metastasis (71, 72). Therefore, the C0 *ZFC3H1^+^* TCs subtype may represent a tumor population with partial stem-like differentiation, potentially regulated under stress or translational control. In addition, the C2 *PRAC1^+^* TCs subtype highly expressed *AREG*, an upstream ligand of *EGFR* that activated the *EGFR* signaling pathway and was frequently associated with tumor proliferation, invasion, and metastasis (73). Moreover, the C4 *BCL2L1^+^* TCs subtype exhibited pronounced heterogeneity, characterized by unique proliferative and metabolic features, establishing it as a key subtype in this study. Its primary overexpressed genes included *NEAT1*, *TMEM59*, *BCL2L1*, *RBP1*, and *PLCB4*. Furthermore, upregulation of *BCL2L1* and *NEAT1* in the liver metastasis-associated subgroup suggested enhanced stress resistance and immune evasion, likely contributing to recurrence and drug resistance. *BCL2L1*, an anti-apoptotic gene (74), has been implicated in tumor progression and therapy resistance in multiple cancers (75–77), potentially promoting tumor survival and metastasis through inhibition of apoptosis (78).

The C4 *BCL2L1^+^* TCs subtype was markedly enriched in OXPHOS, suggesting it may have played a vital role in CRC liver metastasis (79). Notably, this subtype exhibited the lowest differentiation and the highest CytoTRACE stemness score. Further analysis of stemness-related genes revealed high expression of *CTNNB1*, *KDM5B*, and *PROM1*, which together formed a core regulatory network sustaining cancer stem cell properties. Furthermore, CellChat analysis indicated that the CRC microenvironment contained a sophisticated cellular communication network. The C4 TCs subtype was pivotal in the *ADGRE5* and *MK* pathways, likely acting as a regulatory hub within the tumor and immune matrix signaling network. Additionally, B cells were predominantly active in the *MK* pathway, whereas plasma cells were highly expressed in the *ADGRE5* pathway, suggesting that immune cells participated in crosstalk, which may have contributed to immune evasion and tumor progression.

We conducted a comprehensive analysis of the transcriptional regulatory network of the C4 *BCL2L1^+^* TCs subtype in CRC to elucidate its underlying mechanisms. *BCL2L1*, a member of the BCL-2 family, acts as a canonical anti-apoptotic gene, and its elevated expression inhibited mitochondrial pathway-mediated apoptosis, thereby promoting tumor cell survival under stress conditions (80, 81). To define the core regulatory machinery, we identified *ELF1*, *CEBPG*, *KLF3*, *CEBPB*, and *EHF* as the top five TFs with the highest specificity scores in this subtype. Previous studies suggested that *CEBPG* regulated proliferation, migration, and invasion in esophageal cancer cell lines, enhancing these processes upon overexpression and suppressing them after knockdown (82), and that its expression in CRC tumor cells exceeded that in normal colon tissue (59). Consequently, we hypothesized that *BCL2L1* regulation played a pivotal role in CRC liver metastasis. siRNA-mediated knockdown of *CEBPG* significantly reduced cell viability, proliferation, migration, and invasion in SW48 and SNU-81 cells, accompanied by decreased mRNA and protein levels. These results indicated that *CEBPG* critically regulated CRC cell aggressiveness, potentially influencing anti-apoptotic activity and metastatic potential, suggesting its value as a therapeutic target. Furthermore, to identify prognostic molecular indicators, we developed a C4 *BCL2L1^+^* TCs risk score (BTRS) using univariate Cox regression, Lasso regression, and multivariate Cox regression analysis, which effectively stratified patients by risk. Immune microenvironment analysis revealed marked differences between high and low BTRS groups, including upregulation of *SFTPA1*, *SFTPC*, and *NMRK2*, and downregulation of *IGF2*, *MAGEA6*, and *CDH9*. DEGs were enriched in immune modulation, antigen processing, and metabolic pathways. High BTRS patients exhibited increased M0 macrophages and activated CD4 memory T cells, reflecting immune escape and potentially influencing immunotherapy response. These findings may reflect an immune-enriched but functionally constrained tumor microenvironment, where increased immune cell infiltration coexists with impaired effector activity. Drug sensitivity analysis further highlighted differential responses: the low-risk group showed higher CD8^+^ T cells and activated NK cells, whereas the high-risk group displayed elevated CD4^+^ T cells and B cells with reduced NK and dendritic cells, indicating “immune activation but limited function”. High BTRS levels correlated with enhanced immunotherapy responsiveness, supported by upregulated immune checkpoint genes including *TNFSF18*, *HHLA2*, and *CD44*. Moreover, BTRS-based stratification suggested personalized therapeutic strategies, supported by drug sensitivity data for agents such as Gefitinib, Nilotinib, PLX4720, and PD173074, previously applied in EGFR-mutated NSCLC, CML, breast cancer, and gastric cancer (83–85).

We systematically examined the unique molecular characteristics and distribution heterogeneity of the C4 *BCL2L1^+^* TCs subtype in CRC, demonstrating its pivotal role in tumor progression, metabolic regulation, and apoptosis control. Subsequently, we investigated potential clinical translation strategies based on the subtype’s contribution to CRC heterogeneity and treatment resistance, providing valuable guidance for novel therapeutic development. Specifically, we proposed targeted therapies for C4 *BCL2L1^+^* TCs that focused on the critical gene *CEBPG* and its metabolic network. Although TNM staging is widely used to estimate prognosis based on tumor extent, it does not fully reflect biological diversity within tumors or variations in the immune microenvironment. In contrast, the BTRS model incorporates molecular features derived from single-cell analysis and integrates tumor-related transcriptional patterns with immune characteristics. Importantly, BTRS was able to further separate patients with similar clinical stages into different risk categories, indicating that it may offer additional prognostic information beyond conventional staging approaches. Building on this stratification capability, we constructed a BTRS score-based stratified model to facilitate personalized treatment. This model could be integrated with conventional imaging and histopathological evaluation during preoperative assessment and follow-up, thereby supporting clinical decision-making. In addition, combination treatment strategies, such as the co-administration of immunostimulants or metabolic modulators in patients with high BTRS scores, may help enhance therapeutic efficacy and overcome treatment resistance.

While the BTRS risk model showed consistent prognostic stratification in TCGA-derived cohorts, some limitations remain. In particular, the current validation relied mainly on publicly available datasets, which may not fully reflect real-world clinical heterogeneity. Validation in independent, prospectively collected cohorts from a single clinical center would therefore be necessary to better assess the model’s clinical relevance and practical applicability. Although this study is innovative, it presented several limitations. Firstly, although some findings were verified by *in vitro* experiments, since the *in vitro* system cannot fully replicate the *in vivo* TME, animal models are needed to further verify the role of the *CEBPG* regulatory axis in the *BCL2L1^+^*cell subtype. Furthermore, additional protein-level verification of the *CEBPG* downstream regulatory network was required. Immune infiltration was inferred using transcriptome-based deconvolution, which revealed increased M0 macrophages and reduced NK cells in the high-risk group. Although *CEBPG* was identified as a potential regulator of the C4 *BCL2L1^+^* TCs subtype, directly targeting transcription factors remains difficult, largely because they do not possess intrinsic enzymatic activity. To date, there are no reported small-molecule inhibitors specifically designed to target *CEBPG*. Nevertheless, growing studies suggest that oncogenic programs driven by transcription factors may still be amenable to therapeutic intervention through indirect approaches, including modulation of upstream signaling pathways, inhibition of key downstream targets, or interference with transcriptional co-regulators and epigenetic regulators. Moreover, several members of the *BCL2* family, which are closely linked to the biological features of the C4 subtype, have already been successfully targeted in other cancer settings. In conclusion, these findings suggest that indirect targeting of the *CEBPG*-related regulatory network holds promise as a feasible direction for the future treatment development of C4 *BCL2L1*^+^ TCs enriched rectal cancer. Meanwhile, the study lacks *in vivo* validation of the identified regulatory pathways and tumor subtypes. In the future, we will conduct research using animal models to confirm the roles of *CEBPG* and the C4 *BCL2L1^+^* TCs subtype in CRC liver metastasis. Therefore, future research should broaden multi-omics datasets and investigate interactions among immune cells, immune evasion mechanisms, and tolerance processes.

## Conclusion

Using scRNA-seq, this study identified a previously underappreciated CRC tumor cell subtype, C4 *BCL2L1^+^* TCs, which was closely associated with liver metastasis and immune regulatory features. Subsequent *in vitro* experiments further showed that *CEBPG* knockdown inhibited CRC cell proliferation, migration, and invasion, highlighting its potential as a therapeutic target. Based on these results, we constructed a predictive risk score model that integrated genomic data, immune microenvironment features, and drug sensitivity profiles. Overall, the study provides a comprehensive framework and a valuable resource for understanding CRC molecular mechanisms and offers insights that may support future mechanistic studies and precision-oriented therapeutic strategies.

## Data Availability

The single-cell sequencing datasets generated and/or analyzed in this study are publicly accessible in GEO (GSE231559). All original contributions are included in the article and its **Supplementary Materials**, and any inquiries regarding these contributions can be directed to the corresponding authors.

## Supplementary material

Supplementary Figure 1 Analysis of copy number variation of EPCs based on the inference of smooth muscle cells Red indicates amplification, and blue indicates deletion.
