# Supplementary figures and images for: Dissecting tumor heterogeneity in colorectal cancer: uncovering the role of BCL2L1+ cells through single-cell analysis
Source: Front Immunol. 2026 Mar 25;17:1742767. doi: 10.3389/fimmu.2026.1742767 (PMC13056825; doi:10.3389/fimmu.2026.1742767)

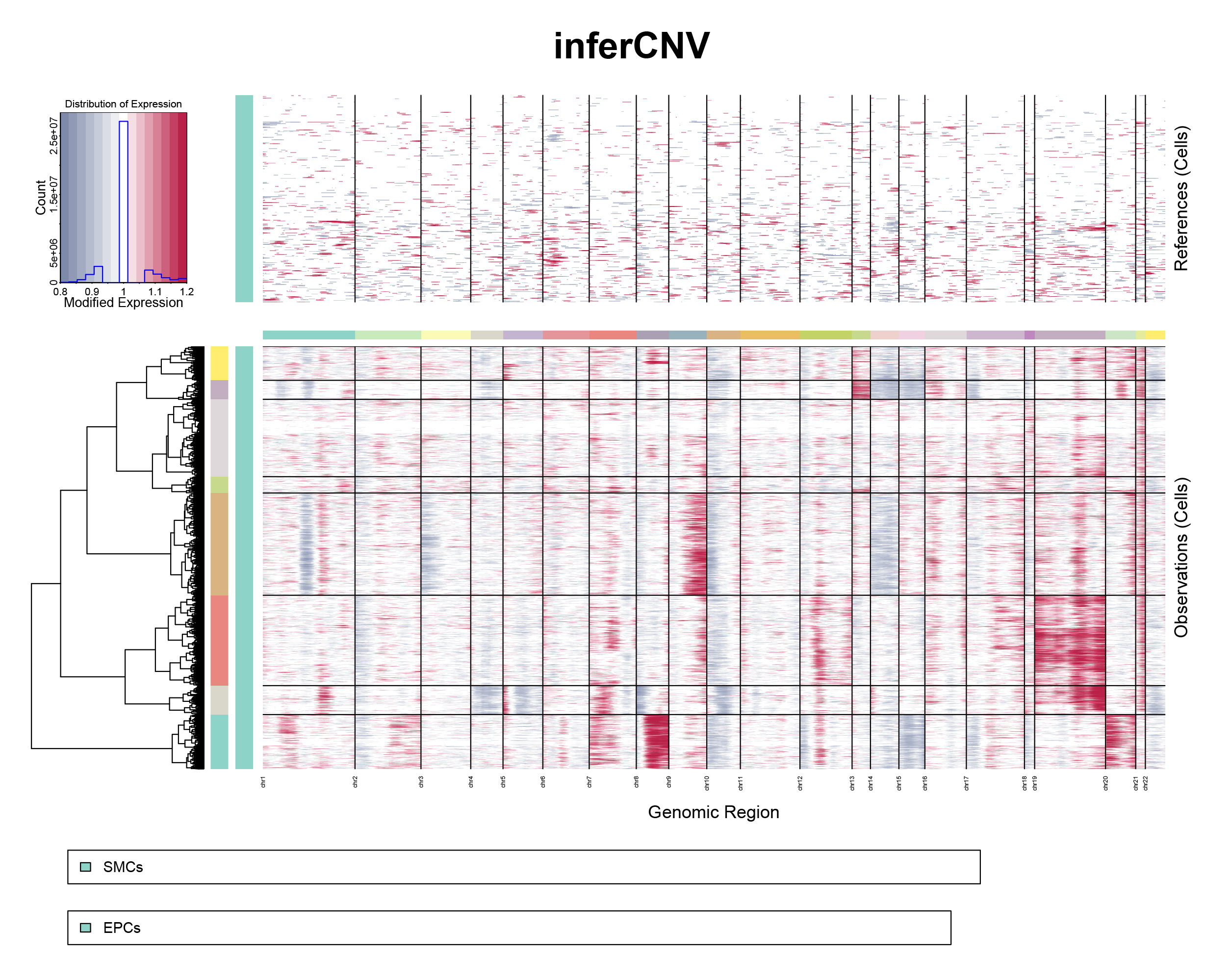

Supplement: Supplementary Figure 1 — Analysis of copy number variation of EPCs based on the inference of smooth muscle cells Red indicates amplification, and blue indicates deletion. [file Image1.jpeg]
